# Ciprofol attenuates the isoproterenol-induced oxidative damage, inflammatory response and cardiomyocyte apoptosis

**DOI:** 10.3389/fphar.2022.1037151

**Published:** 2022-11-22

**Authors:** Yunzhao Yang, Zhongyuan Xia, Cheng Xu, Chunchun Zhai, Xi Yu, Siqi Li

**Affiliations:** Department of Anesthesiology, Renmin Hospital of Wuhan University, Wuhan, China

**Keywords:** ciprofol, isoproterenol, oxidative stress, inflammation, apoptosis

## Abstract

**Background and Purpose:** Ciprofol (HSK3486), a novel 2,6-disubstituted phenol derivative, is a new intravenous anesthetic compound with a similar chemical structure to propofol. Animal studies have also shown that propofol plays a protective role in a variety of cardiovascular diseases, including myocardial infarction, myocardial ischemia-reperfusion injury and takotsubo syndrome. However, whether ciprofol exerts cardioprotective effects on myocardial infarction remains unclear. Thus, the aim of this work was to explore the potential cardioprotective mechanism of ciprofol on isoproterenol (ISO)-induced myocardial infarction.

**Experimental Approach:** In the present study, male C57BL/6 mice were subjected to subcutaneous injection of ISO (100 mg/kg) for 2 consecutive days to induce experimental myocardial infarction. Herein, we found that ciprofol could inhibit the abnormal increase in myocardial injury enzymes, the area of myocardial infarction and cardiac dysfunction in ISO-treated mice. Ciprofol administration increased the activity of superoxide dismutase and reduced the levels of NADPH oxidase and malondialdehyde in ISO-treated hearts. Additionally, ciprofol administration markedly reduced the expression of pro-inflammatory cytokines and cardiomyocyte apoptosis. In an *in vitro* model, the results also confirmed that ciprofol could inhibit ISO-induced oxidative damage, the inflammatory response and cardiomyocyte apoptosis. Moreover, ciprofol can activate the sirtuin1 (Sirt1)/nuclear factor erythroid 2-related factor 2 (Nrf2) pathway and Sirt1 and Nrf2 inhibition almost abolished ciprofol-mediated cardioprotective effects.

**Interpretation:** Ciprofol protects the heart against ISO-induced myocardial infarction by reducing cardiac oxidative stress, the inflammatory response and cardiomyocyte apoptosis.

## Introduction

Cardiovascular diseases have been one of the leading causes of death and disability in the world and are a major contributor to the decline in quality of life (Podolec and Matusik, 2019; Wang et al., 2021a; Schooling, 2022). Epidemiological data showed that 17.8 million people worldwide have died of cardiovascular diseases every year, corresponding to 330 million years of life loss and 35.6 million years of disabled life (GBD, 2018; Mensah et al., 2019; Shi et al., 2022). Myocardial infarction is the most lethal manifestation of cardiovascular diseases, characterized by the reduction of blood perfusion in the heart, which leads to a decrease in the oxygen supply of the heart and then results in myocardial damage involving both necrosis and apoptosis of the heart muscle (de Lemos et al., 2019; Roumeliotis et al., 2021; Huse et al., 2022; Lan et al., 2022; Wang et al., 2022). Since cardiomyocytes are terminally differentiated cells and do not regenerate, preventing cardiomyocyte injury and loss is a promising strategy for myocardial infarction.

Ciprofol (HSK3486), a novel 2,6-disubstituted phenol derivative, is a new intravenous anesthetic compound with a similar chemical structure to propofol (Hu et al., 2021a; Liu et al., 2021a). Ciprofol has the pharmacodynamic characteristics of a rapid rate of onset and rapid recovery and is intended for use to induce anesthesia in endoscopy and intensive care unit (ICU) patients in pre-clinical experiments (Liu et al., 2021a; Long et al., 2022). Recent studies have shown that ciprofol has good efficacy and safety, and may reduce anesthesia-related hemodynamic inhibition when compared with propofol during colonoscopy (Teng et al., 2021). Animal studies have also shown that propofol plays a protective role in a variety of cardiovascular diseases, including myocardial infarction, myocardial ischemia-reperfusion injury and takotsubo syndrome (Oras et al., 2017; Zhang et al., 2021a; Li et al., 2022). However, whether ciprofol exerts cardioprotective effects on myocardial infarction remains unclear.

In the present study, we identified and characterized the role of ciprofol in myocardial infarction and its underlying mechanism. The results showed that ciprofol protects the heart against isoproterenol (ISO)-induced myocardial infarction by reducing cardiac oxidative stress, inflammatory response and cardiomyocyte apoptosis. Overall, our results provide new insight into the protective mechanism of ciprofol against myocardial infarction.

## Materials and methods

### Animals and experimental design

Eighty male C57BL/6 mice (20–24 g, 8–10 weeks) were purchased from Gempharmatech Co., Ltd. (Jiangsu, China). All animals were housed under standard barrier conditions with controlled temperature (21°C–24°C) and humidity (50%–65%) on a 12-h light/dark cycle and had free access to a standard rodent diet and water. The mice were subcutaneously injected with ISO (100 mg/kg, I5627, purchased from Sigma-Aldrich, United States) for 2 consecutive days to induce experimental myocardial infarction, as reported previously (Cheng et al., 2015; Lu et al., 2018). In addition, an Alzet 1003D osmotic pump (Cupertino, United States) filled with ciprofol (100 μl, purchased from Haisco Pharmaceutical Group Co., Ltd., China) or equivalent normal saline (NS) was implanted into the abdomen of mice 1 h before ISO treatment. At 24 h after the last ISO injection, cardiac functions were evaluated and the animals were then euthanized (200 mg/kg pentobarbital sodium, i. p.) for collection of blood and hearts. All animal care and experimental procedures were conducted according to the Guide for the Care and Use of Laboratory Animals (NIH published) and approved by the Animal Care and Use Committee of Renmin Hospital of Wuhan University (WDRM-20200713).

### Echocardiography and hemodynamics

Cardiac functions were examined by echocardiography using a Vevo 2100 imaging system (VisualSonics, Canada) as previously described (Yin et al., 2022). In brief, mice were lightly sedated with 2% isoflurane, and left ventricle (LV) geometry was assessed at the midpapillary muscle level. The echocardiographic parameters, including LV end-systolic diameter (LVESD), LV end-diastolic diameter (LVEDD), ejection fraction (EF%) and fractional shortening (FS%) were recorded and measured. Then, a microtip catheter transducer (Millar, Inc., United States) was inserted into the right carotid artery and advanced into the left ventricle, the signals were continuously recorded using a Millar PressureVolume system (Millar, Inc., United States). The hemodynamics parameters, the maximal slope of the systolic pressure increment (+dP/dt max) and diastolic pressure decrement (-dP/dt max), left ventricular systolic pressure (LVSP) and left ventricular end-diastolic pressure (LVEDP) were recorded and measured.

### 2,3,5-Triphenyltetrazolium chloride staining

After echocardiography analysis, the mice were euthanized and the hearts were obtained and then washed with cold phosphate buffered saline. The hearts were frozen at −30°C for 30 min, carefully sliced and then incubated with 1% Triphenyltetrazolium chloride (TTC) solution (T8877, Sigma-Aldrich, United States) at 37°C. The infarct volume for each brain was calculated using the following formula: infarct volume ratio (%) = total infarct volume/total volume of hearts × 100%.

### Myocardial injury determination

Following the determination of cardiac function, blood samples were collected and centrifuged to obtain serum. Myocardial injury indexes of lactate dehydrogenase (LDH, A020-2-2), creatine kinase isoenzymes (CK-MB, E006-1-1) and cardiac troponin T (cTnT, H149-4) were determined by a commercial kit purchased from Nanjing Jiancheng Bioengineering Institute (Jiangsu, China).

### Oxidative stress assay

To detect cardiac oxidative damage, dihydroethidium (DHE, ab145360, Abcam, United States) staining was performed, as reported previously (Jiang et al., 2021). Briefly, the heart was excised transversely into 4- to 5-μm slides, incubated with 10 μmol/L DHE and then examined under a fluorescence microscope (IX73, OLYMPUS, Japan). In addition, the activity of superoxide dismutase (SOD, S0101 M) and the levels of NADPH oxidase (NOX, S0179) and malondialdehyde (MDA, S0131M) in the hearts and H9c2 cells were detected by a commercial kit purchased from Beyotime Biotechnology (Shanghai, China).

### TdT-mediated dUTP nick-end labeling staining

The hearts were fixed in 10% paraformaldehyde, embedded in paraffin and then excised transversely into 4- to 5-μm slides. TUNEL staining was performed using Click-iT™ Plus TdT-mediated dUTP nick-end labeling (TUNEL) Assay Kits (C10617, Thermo Fisher Scientific, United States) according to the manufacturer’s instructions. For the H9c2 cells, TUNEL staining was performed as previously described (Dong et al., 2021).

### Cell culture and treatment

H9c2 cells were purchased from the China Center for Type Culture Collection (CCTCC, GDC0606, China) and cultured in Dulbecco’s modified Eagle’s medium (DMEM, 12100046, Gibco, United States) in a humidified atmosphere of 95% O_2_ and 5% CO_2_ at 37°C (Zhou et al., 2022). After overnight starvation, H9c2 cells were incubated with ISO (10 μM) for 24 h to mimic ISO-induced cardiomyocyte injury. In addition, H9c2 cells were pretreated with ciprofol (5 μM) before ISO insult for 6 h and the dose of ciprofol was determined according to our preliminary experimental results. To verify the hypothesis that ciprofol provided protection *via* activation of Sirt1 and Nrf2, cells were subjected to Sirt1 inhibitors EX527 (1 μmol/L) or Nrf2 inhibitors ML385 (1 μmol/L) at 1 h before DOX administration.

### Quantitative real-time RT-PCR

Total RNA was obtained from frozen heart tissue and H9c2 cells then reverse-transcribed into cDNA using High Capacity cDNA Reverse Transcription Kit (4368814, Applied Biosystems, United States) (Zhou et al., 2021). Real-time PCR was performed using a One Step TB Green® PrimeScript™ RT-PCR Kit II (RR086A, Takara, Japan) and the mRNA levels of genes were determined with the 2^-△△Ct^ method. Glyceraldehyde-3-phosphate dehydrogenase (GAPDH) was selected as the internal control and all details about the primers are presented in Table 1.

**TABLE 1:** Primer sequences for RT-PCR assays.

Gene	Species		Sequence (5’-3’)
IL-6	Mouse	Forward	AGT​TGC​CTT​CTT​GGG​ACT​GA
		Reverse	TCC​ACG​ATT​TCC​CAG​AGA​AC
IL-6	Rat	Forward	GTT​GCC​TTC​TTG​GGA​CTG​ATG
		Reverse	ATA​CTG​GTC​TGT​TGT​GGG​TGG​T
IL-17A	Mouse	Forward	TCC​AGA​AGG​CCC​TCA​GAC​TA
		Reverse	AGC​ATC​TTC​TCG​ACC​CTG​AA
IL-17A	Rat	Forward	ATC​CCT​CAA​AGT​TCA​GTG​TGT​CC
		Reverse	GGA​CAA​TAG​AGG​AAA​CGC​AGG​T
TNF-α	Mouse	Forward	CCC​AGG​GAC​CTC​TCT​CTA​ATC
		Reverse	ATG​GGC​TAC​AGG​CTT​GTC​ACT
TNF-α	Rat	Forward	CTA​CTC​CCA​GGT​TCT​CTT​CAA
		Reverse	GCT​GAC​TTT​CTC​CTG​GTA​TGA
GAPDH	Mouse	Forward	AAC​TTT​GGC​ATT​GTG​GAA​GG
		Reverse	CAC​ATT​GGG​GGT​AGG​AAC​AC
GAPDH	Rat	Forward	GAA​GGT​CGG​TGT​GAA​CGG​ATT​TG
		Reverse	CAT​GTA​GAC​CAT​GTA​GTT​GAG​GTC​A

### Western blotting

Total protein was obtained from frozen heart tissue and H9c2 cells, the cytosolic and nuclear proteins were isolated using a commercial kit (78833, Thermo Fisher Scientific, United States) as described in our previous study (Hu et al., 2021b). Then, total protein was loaded into 10% SDS-PAGE gels, electro-transferred to an Immobilon-P membrane (IPVH00010, Millipore, China) and incubated with the primary antibodies against cTnI (ab52862, Abcam, United States), p-cTnI (ab58546, Abcam, United States), SOD1 (ab13498, Abcam, United States), SOD2 (ab13533, Abcam, United States), Sirt1 (ab189494, Abcam, United States), p-Nrf2 (ab76026, Abcam, United States), Nrf2 (ab62352, Abcam, United States), heme oxygenase-1 (HO-1, ab68477, Abcam, United States), Lamin B1 (ab16048, Abcam, United States) and GAPDH (ab8245, Abcam, United States). After incubation with the secondary antibody, the membranes were scanned using an Odyssey infrared imaging system (LI-COR, United States), and GAPDH was selected as the internal control.

### Statistical analysis

All results are shown as the means ± standard deviation (SD), and the statistical significance between groups was determined using one-way ANOVA followed by a post hoc Tukey’s test. Differences were deemed significant at *p* < 0.05.

## Results

### Ciprofol treatment suppressed ISO-induced myocardial damage

**FIGURE 1:**
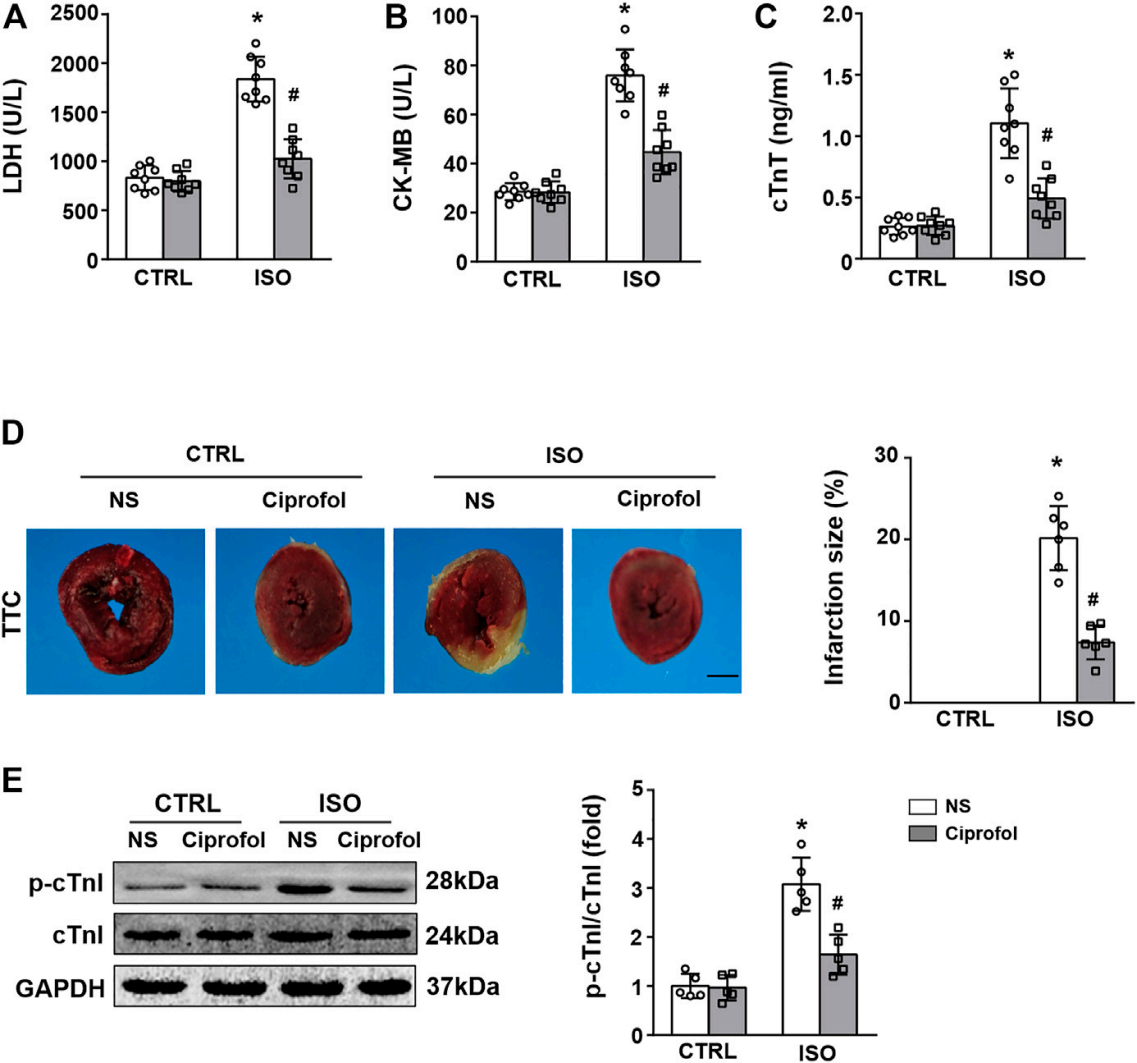
Ciprofol treatment suppressed ISO-induced myocardial damage. CK-MB **(A)**, LDH **(B)** and cTnT **(C)** activities in each group were detected (*n* = 8) **(D)** TTC staining in each group was detected. (*n* = 6). **(E)** Western blotting analysis of p-cTnI, and cTnI in the heart (*n* = 5). ∗*p* < 0.05 compared to the CTRL group. ^#^ *p* < 0.05 compared to the ISO group.

As shown in Figures 1A–C, ISO insults increased the content of serum CK-MB, LDH, and cTnT, and these pathological alterations were attenuated by ciprofol treatment. The TTC staining results also showed that ciprofol treatment suppressed ISO-induced myocardial infarction (Figure 1D). The results of western blots also showed that ciprofol treatment decreased the phosphorylation of cTnI in the hearts after ISO insults (Figure 1E).

### Ciprofol treatment improved ISO-induced cardiac dysfunction

**FIGURE 2:**
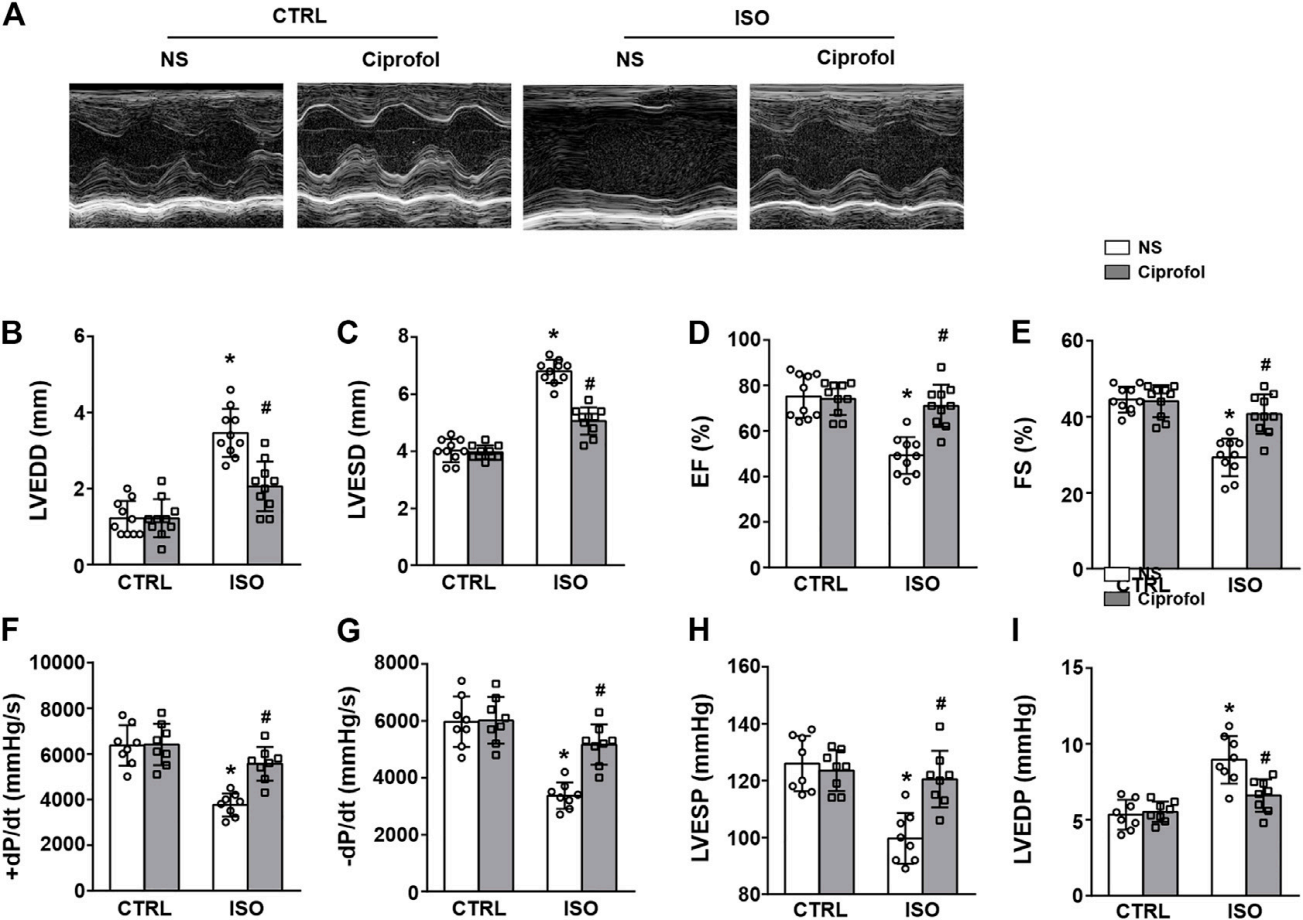
Ciprofol treatment improved ISO-induced cardiac dysfunction. **(A)** Echocardiography images from each group. LVEDD **(B)**, LVESD **(C)**, LVEF **(D)** and LVFS **(E)** in each group were measured (*n* = 10). +dP/dt **(F)**, -dP/dt **(G)**, LVESP **(H)** and LVEDP **(I)** in each group were measured (*n* = 8). ∗*p* < 0.05 compared to the CTRL group. ^#^ *p* < 0.05 compared to the ISO group.

As shown in Figures 2A–E, ISO insults led to obvious cardiac dysfunction, as indicated by the increased LVEDD and LVESD, and the reduced EF (%) and FS (%). However, ciprofol treatment significantly improved ISO-induced LV systolic and diastolic dysfunction (Figures 2A–E). Similar results were obtained with invasive hemodynamic measurements (Figures 2F–I).

### Ciprofol treatment attenuated ISO-induced oxidative stress

**FIGURE 3:**
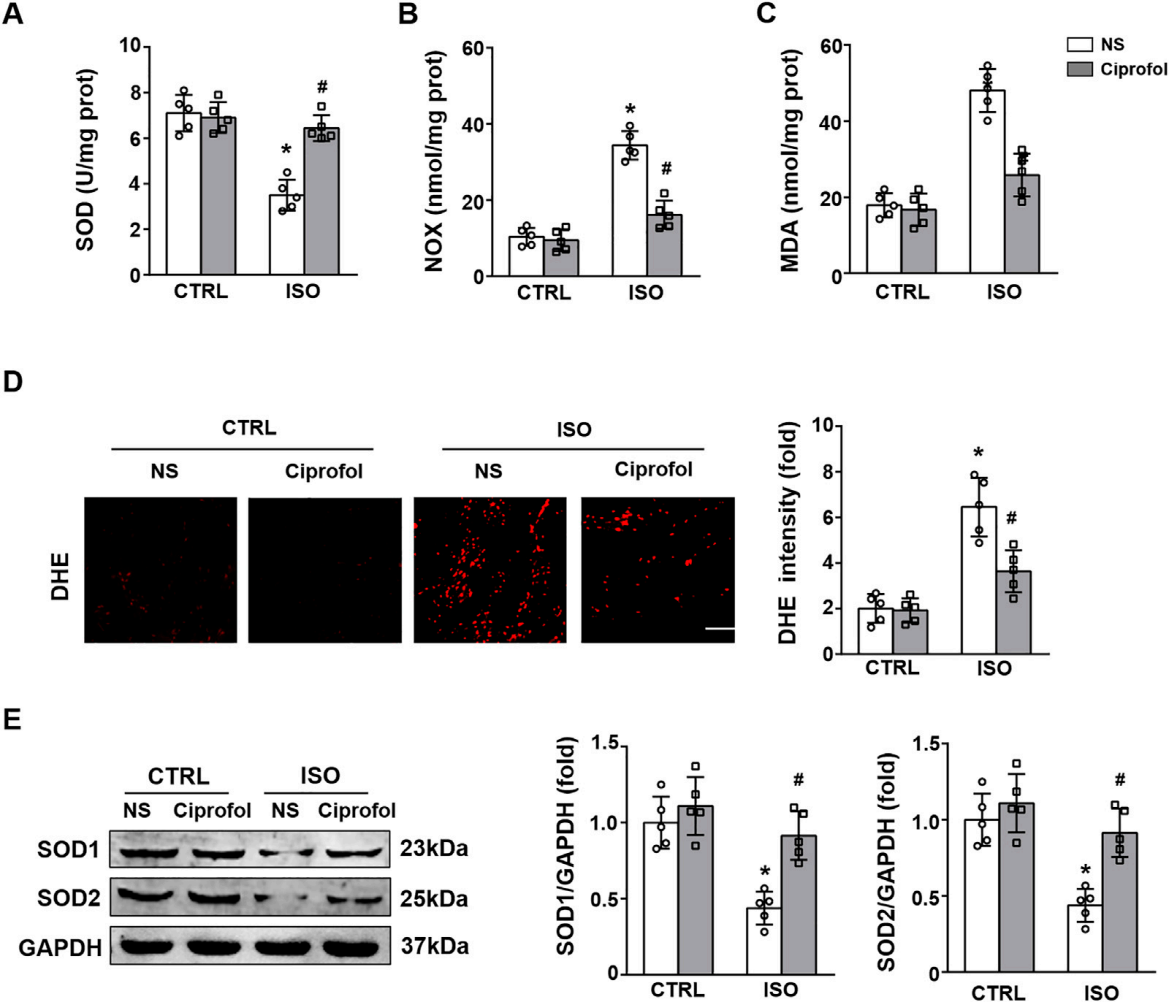
Ciprofol treatment attenuated ISO-induced oxidative stress. SOD **(A)**, NOX **(B)** and MDA **(C)** levels in each group were assessed (*n* = 5). **(D)** DHE staining and the quantitative results (*n* = 5; scale bar, 50 μm) **(E)** Western blotting analysis of SOD1, and SOD2 in the heart (*n* = 5). ∗*p* < 0.05 compared to the CTRL group. ^#^ *p* < 0.05 compared to the ISO group.

The results showed that ISO insults reduced the activity of SOD and increased the levels of NOX and MDA (Figures 3A–C). However, these pathological changes were attenuated by ciprofol treatment (Figures 3A–C). The DHE staining results showed that ciprofol treatment attenuated ISO-induced oxidative stress (Figure 3D). In addition, ciprofol treatment also increased SOD1 and SOD2 expression in ISO-treated hearts (Figure 3E).

### Ciprofol treatment inhibited ISO-induced inflammation and cardiomyocyte apoptosis

**FIGURE 4:**
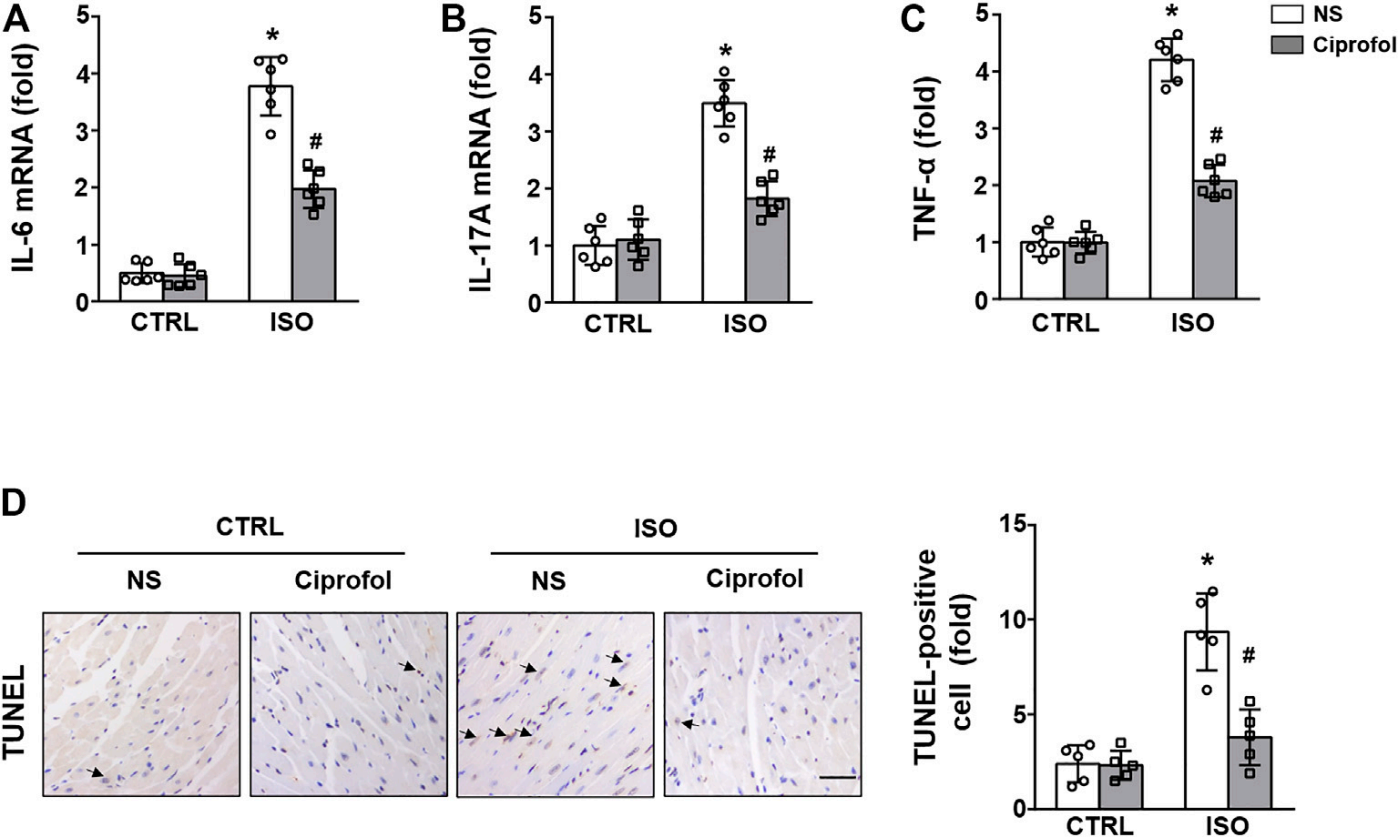
Ciprofol treatment inhibited ISO-induced inflammation and cardiomyocyte apoptosis. The mRNA levels of IL-6 **(A)**, IL-17A **(B)** and TNF-α **(C)** were measured (*n* = 6). **(D)** Representative images of TUNEL staining in each group (*n* = 5). ∗*p* < 0.05 compared to the CTRL group. ^#^ *p* < 0.05 compared to the ISO group.

As shown in Figures 4A–C, the expression levels of the proinflammatory cytokines IL-6 IL-17 and TNF-α were markedly elevated after ISO insults. However, ciprofol treatment largely suppressed these increases in IL-6 IL-17 and TNF-α expression. The TUNEL staining results also revealed significant cardiomyocyte apoptosis in the ISO group, and ciprofol treatment significantly attenuated ISO-induced cardiomyocyte apoptosis (Figure 4D).

### Ciprofol treatment activated the Sirt1/Nrf2 signaling pathway

**FIGURE 5:**
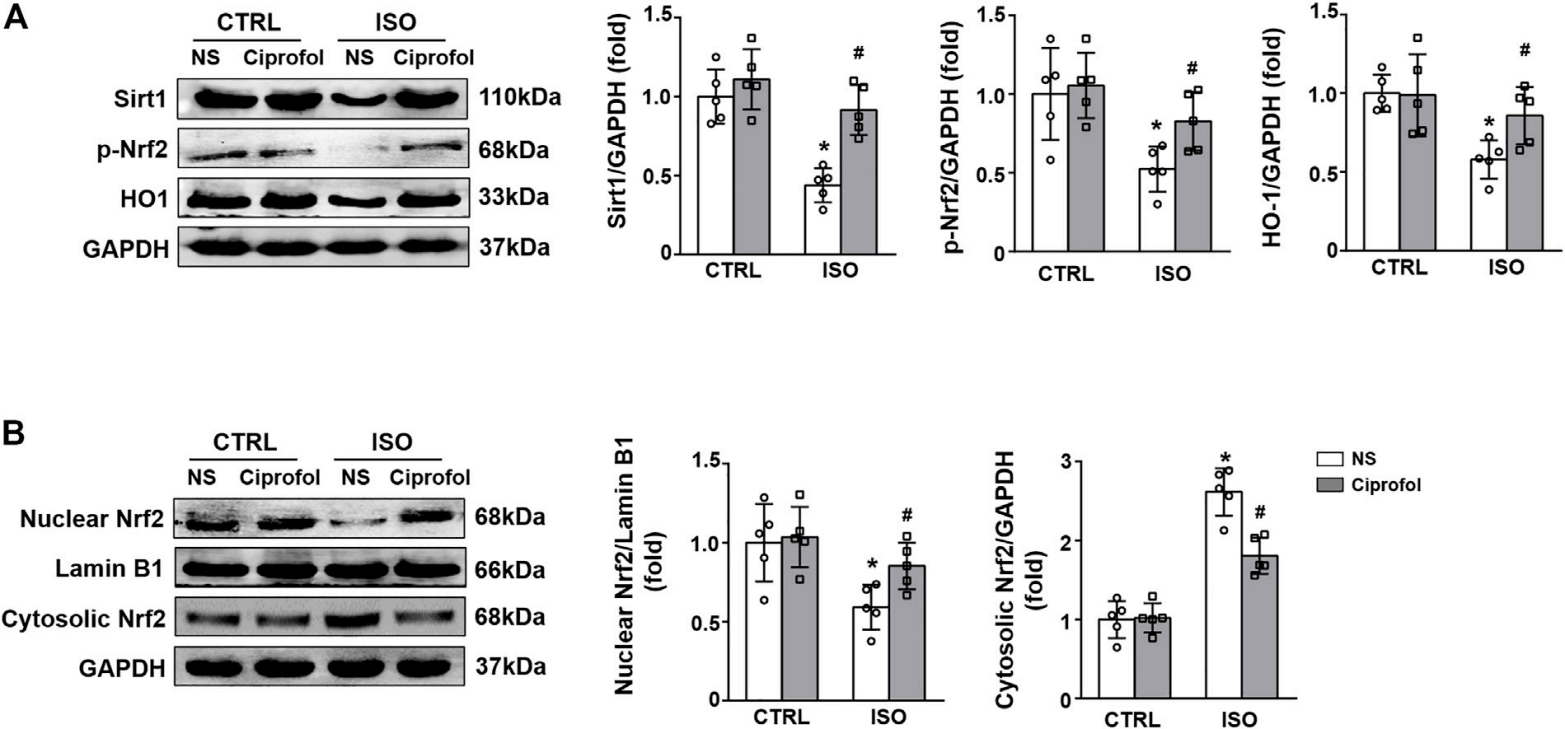
Ciprofol treatment activated the Sirt1/Nrf2 signaling pathway. **(A)** Western blotting analysis of Sirt1, p-Nrf2 and HO-1 in the hearts (*n* = 5). **(B)** Western blotting analysis of nuclear Nrf2 and cytosolic Nrf2 in the hearts (*n* = 5). ^∗^ *p* < 0.05 compared to the CTRL group. ^#^ *p* < 0.05 compared to the ISO group.

We next detected the effect of ciprofol on Sirt1 and Nrf2 expression. As shown in Figure 5A, ISO insults decreased myocardial Sirt1, p-Nrf2 and HO-1 expression, and this effect was prevented by ciprofol treatment. In addition, ciprofol treatment significantly increased Nrf2 expression in the nucleus while decreasing it in the cytoplasm in in ISO-treated hearts (Figure 5B).

### Ciprofol treatment blunted ISO-induced myocardial damage and oxidative stress *in vitro*

**FIGURE 6:**
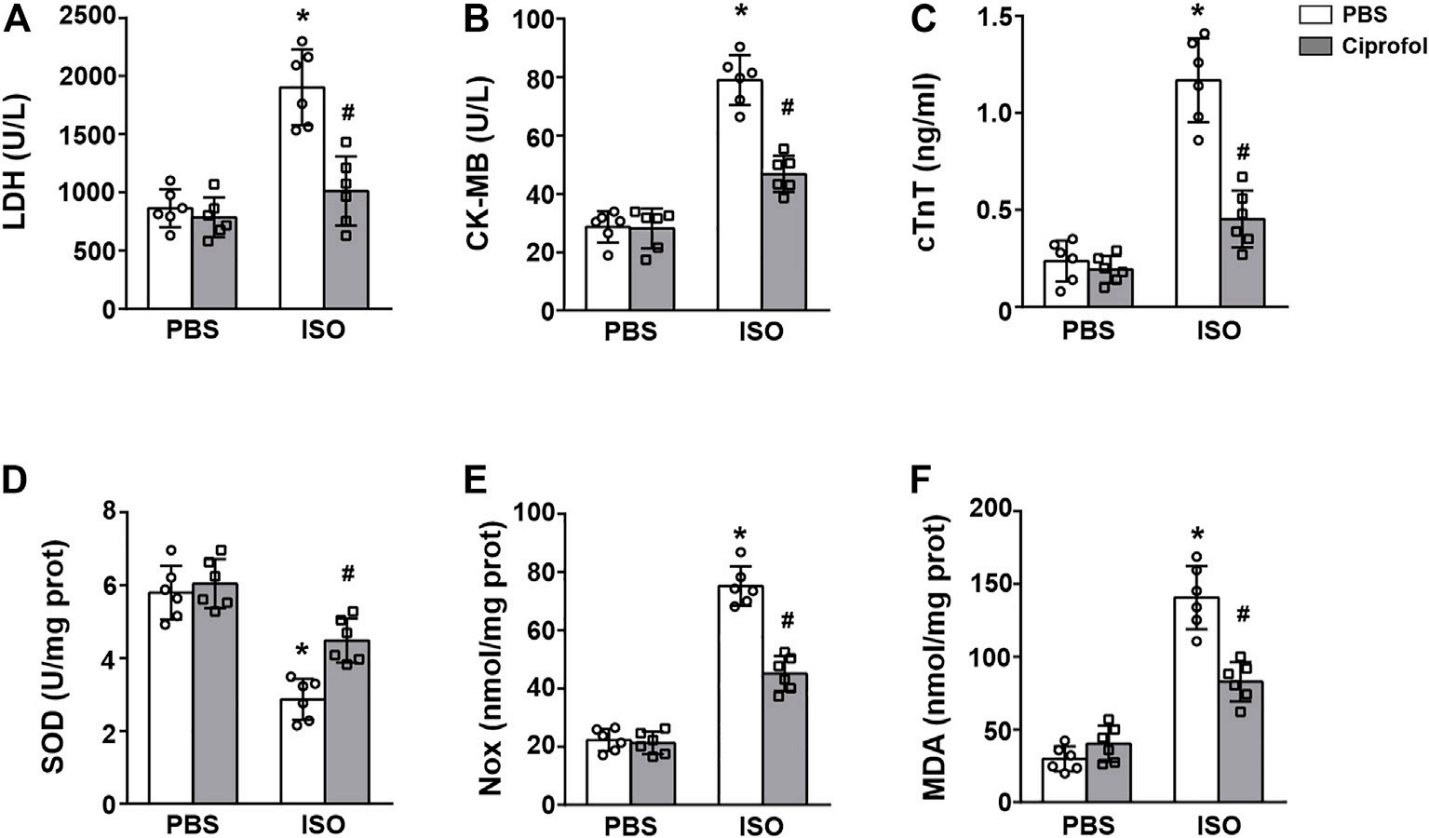
Ciprofol treatment blunted ISO-induced myocardial damage and oxidative stress *in vitro*. CK-MB **(A)**, LDH **(B)** and cTnT **(C)** activities in each group were detected (*n* = 6). SOD **(C)**, NOX **(D)** and MDA **(E)** levels in each group were assessed (*n* = 6). ∗*p* < 0.05 compared to the PBS group. ^#^ *p* < 0.05 compared to the ISO group.

We next detected the cardioprotective effect of ciprofol *in vitro*. The results showed that the contents of CK-MB, LDH and cTnT were increased in ISO-treated H9c2 cells, and these pathological alterations could be blunted by ciprofol treatment (Figures 6A–C). In addition, ciprofol treatment markedly restored the activity of SOD and reduced myocardial NOX and MDA levels in ISO-treated cells (Figures 6D–F).

### Ciprofol treatment blunted ISO-induced inflammation and cardiomyocyte apoptosis *in vitro*

**FIGURE 7:**
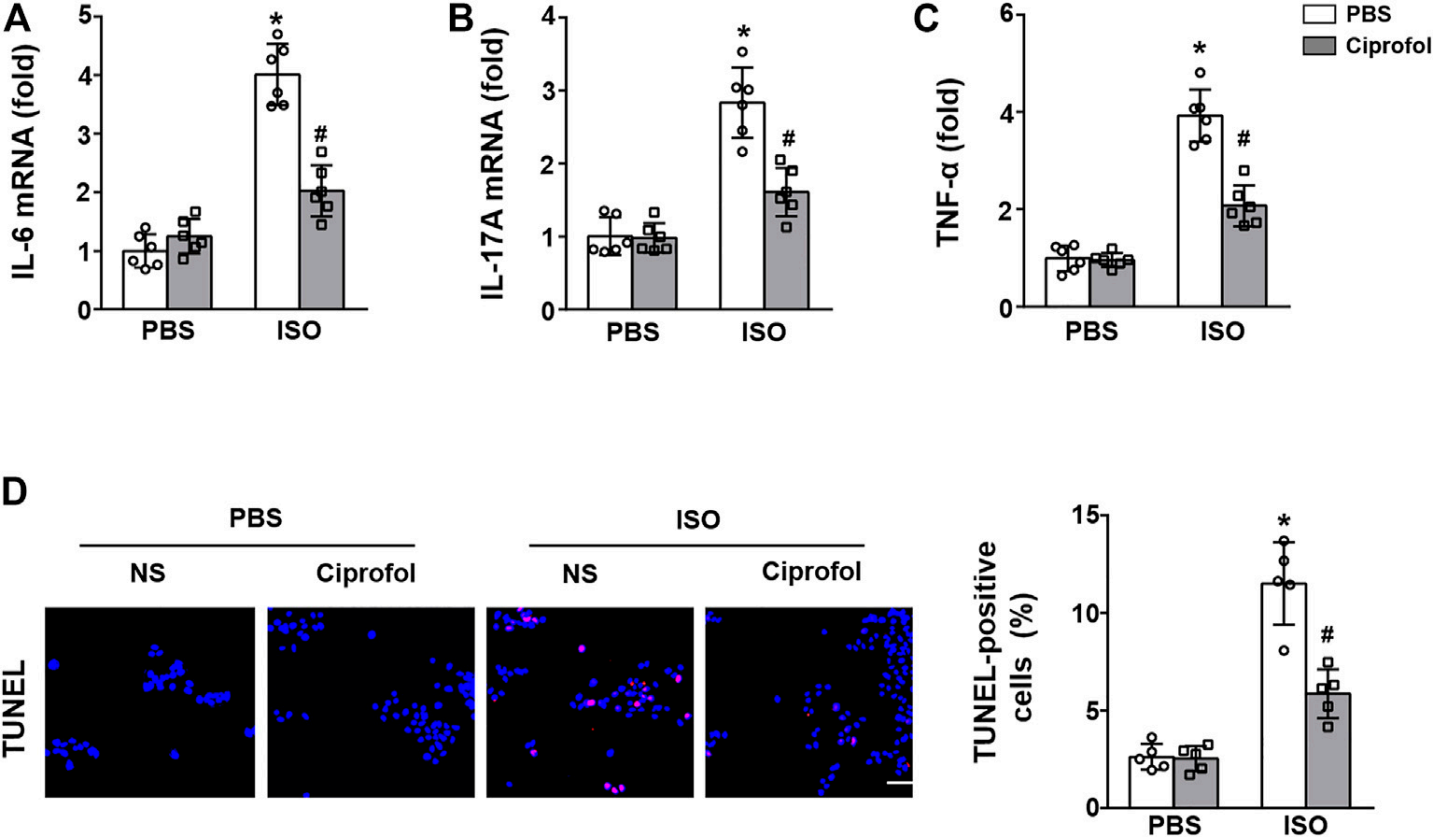
Ciprofol treatment blunted ISO-induced inflammation and cardiomyocyte apoptosis *in vitro*. The mRNA levels of IL-6 **(A)**, IL-17A **(B)** and TNF-α **(C)** were measured (*n* = 6). **(D)** Representative images of TUNEL staining in each group (*n* = 5). ∗*p* < 0.05 compared to the PBS group. ^#^ *p* < 0.05 compared to the ISO group.

As shown in Figures 7A–C, the mRNA expression levels of IL-6 IL-17 and TNF-α were markedly elevated in ISO-treated H9c2 cells, and these pathological alterations could be blunted by ciprofol treatment. Consistent with the *in vivo* study, the TUNEL staining results also showed that ciprofol treatment attenuated ISO-induced cardiomyocyte apoptosis *in vitro* (Figure 7D).

### Sirt1 and Nrf2 inhibition abolished the cardioprotective and anti-oxidative stress effects of ciprofol *in vitro*

**FIGURE 8:**
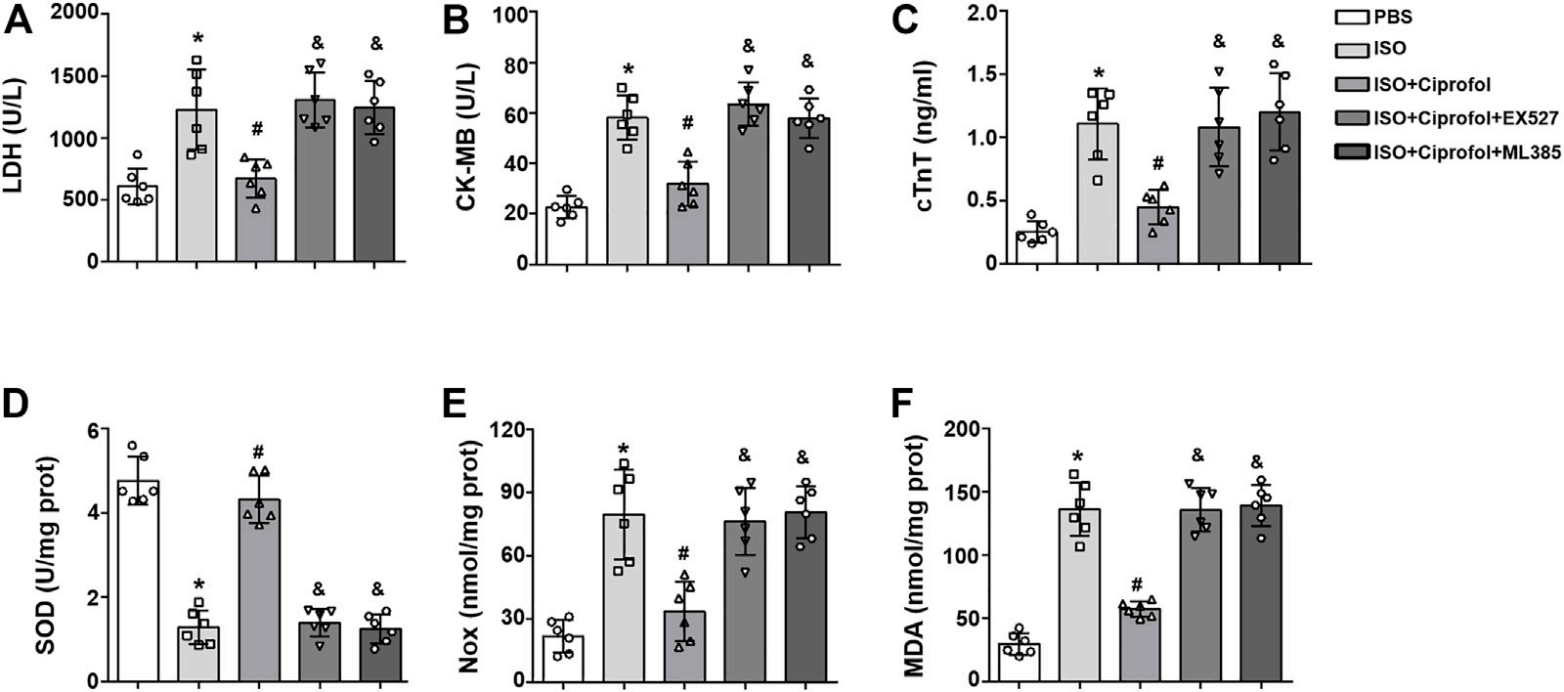
Sirt1 and Nrf2 inhibition abolished the cardioprotective and anti-oxidative stress effects of ciprofol *in vitro*. CK-MB **(A)**, LDH **(B)** and cTnT **(C)** activities in each group were detected (*n* = 6). SOD **(C)**, NOX **(D)** and MDA **(E)** levels in each group were assessed (*n* = 6). ∗*p* < 0.05 compared to the PBS group. ^#^ *p* < 0.05 as compared to the ISO group. ^&^ *p* < 0.05 compared to the ISO + ciprofol group.

As shown in Figure 8A, Sirt1 and Nrf2 inhibitors increased the content of CK-MB, LDH and cTnT in ISO-treated H9c2 cells and abolished the cardioprotective effects of ciprofol (A–C). In addition, Sirt1 and Nrf2 inhibitors offset the ciprofol-mediated anti-oxidative stress, as indicated by the reduced the activity of SOD and increased myocardial NOX and MDA levels (D–F).

### Sirt1 and Nrf2 inhibition abolished the anti-inflammation and anti-apoptosis effects of ciprofol *in vitro*

**FIGURE 9:**
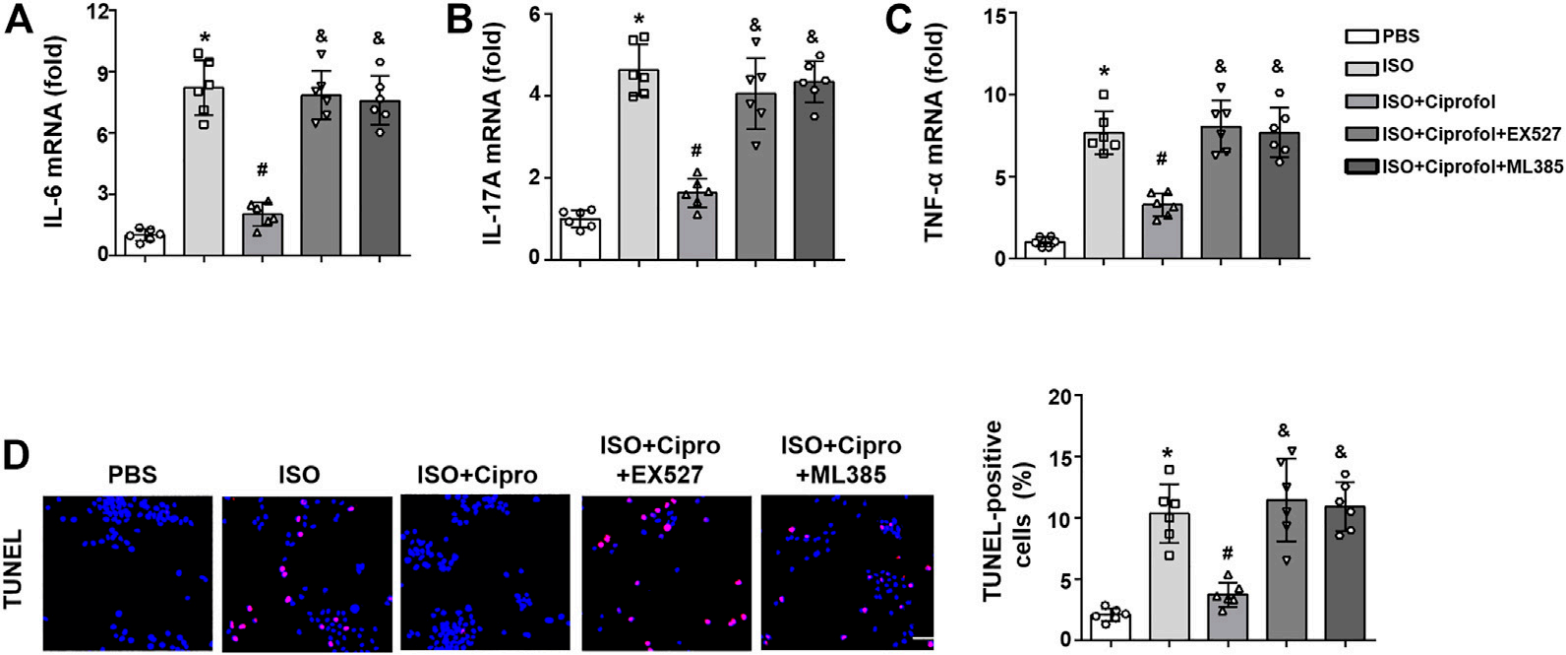
Sirt1 and Nrf2 inhibition abolished the anti-inflammation and anti-apoptosis effects of ciprofol *in vitro*. The mRNA levels of IL-6 **(A)**, IL-17A **(B)** and TNF-α **(C)** were measured (*n* = 6). **(D)** Representative images of TUNEL staining in each group (*n* = 5). ∗*p* < 0.05 compared to the PBS group. ^#^ *p* < 0.05 compared to the ISO group. ^&^ *p* < 0.05 compared to the ISO + ciprofol group.

The results showed that Sirt1 and Nrf2 inhibitors offset the ciprofol-mediated effects against inflammation, as evidenced by increased mRNA expression levels of IL-6 IL-17 and TNF-α (Figures 9A–C). In addition, the TUNEL staining results also showed that Sirt1 and Nrf2 inhibitors also offset the ciprofol-mediated anti-apoptosis *in vitro* (Figure 9D).

## Discussion

ISO is a synthetic nonselective β-adrenergic receptor (β-AR) agonist and is mainly used in the treatment of bradycardia, atrioventricular block and cardiac arrest by exerting positive chronotropic and inotropic effects (Abdelzaher et al., 2021; Liu et al., 2021b; Coquerel et al., 2021). However, high dose of ISO can increase myocardial oxygen consumption, induce heart stress, and result in the development of myocardial injury and infarction-like changes (Brooks and Conrad, 2009; Allawadhi et al., 2018; Al-Botaty et al., 2022). In experimental animals, ISO induces multiple pathological changes in the heart that are associated with oxidative damage and the generation of inflammatory and apoptotic responses, which are similar to those observed in acute myocardial infarction in humans (Wong et al., 2017; Halade and Lee, 2022; Mu et al., 2022). Based on these advantages, it is used as a standard experimental model to detect the preventive and the protective effects against myocardial ischemia and infarction. In line with previous studies, we also found a high dose of ISO stimulation induced an abnormal increase in myocardial injury enzymes, severe myocardial infarction and cardiac dysfunction. These results indicated that we successfully induced a myocardial infarction mouse model with ISO insults.

As a novel anesthetic compound similar to propofol, ciprofol has the pharmacodynamic characteristics of a rapid rate of onset and rapid recovery and an equivalent efficacy ratio to propofol of 1/4 to 1/5 (Hu et al., 2021a; Liu et al., 2021a). Emerging data have shown that propofol and its analogues exerts profound protective effects on variety of cardiovascular diseases (Oras et al., 2017; Zhang et al., 2021a; Li et al., 2022). Li et al. reported that propofol pretreatment protects myocardium from ischemia/reperfusion injury by reducing oxidative stress and ferroptosis (Oras et al., 2017; Zhang et al., 2021a; Li et al., 2022). Fropofol treatment prevented hypertrophy, cardiac dysfunction, and disease progression in mice with hypertrophic cardiomyopathy (Huang et al., 2020). However, whether ciprofol exerts cardioprotective effects on myocardial infarction remains unclear. Thus, we evaluated the effect of ciprofol on ISO-induced myocardial infarction. As expected, ciprofol treatment inhibited the abnormal increase in CK-MB, LDH and cTnT and reduced infarction size. Furthermore, ciprofol treatment significantly improved ISO-induced LV systolic and diastolic dysfunction, which again proved that ciprofol was a myocardial protector.

Although the pathogenesis of myocardial infarction is complex, oxidative stress has been proven to be crucial in the occurrence and development of myocardial infarction (Hori and Nishida, 2009; Xiang et al., 2021; Luo et al., 2022). Oxidative stress reflects an imbalance between reactive oxygen species (ROS) and the anti-oxidant system, and cardiomyocytes can produce a large amount of ROS under ischemia and hypoxia (Hou et al., 2021; Rasool et al., 2021). Previous research has demonstrated that ISO can induce excessive production of ROS and lipid peroxidation, and overproduced ROS are capable of attacking cells, causing cell injury and death (Farag et al., 2021; Wu et al., 2021). Due to the lack of effective anti-oxidant defense, cardiomyocytes are especially vulnerable to oxidative stress, which is closely related to the development of myocardial infarction (Xiang et al., 2021). Thus, suppressing oxidative stress has been proven to be an effective strategy to prevent myocardial infarction.

SOD is a key component in cellular anti-oxidation systems, which can catalyze and scavenge ROS (Qiao et al., 2021). NOX are an important source of ROS generation and MDA has been described as a lipid peroxidation product (Wang et al., 2021b; Zhang et al., 2021b). As expected, ISO insults dramatically suppressed the activities of SOD and increased the generation of MDA and NOX, and these pathological changes were partially restored by ciprofol treatment. In addition, DHE staining was performed to detect cardiac ROS levels. Our results also showed that DHE expression in the hearts of ciprofol-treated mice was significantly decreased after ISO insults, directly reflecting that ciprofol could reduce cardiac ROS generation during myocardial infarction.

The inflammatory response has been proven to be a key feature of myocardial infarction and contributes to cardiac damage and cardiomyocyte apoptosis (Thackeray et al., 2018; Bai et al., 2021). Emerging studies have identified that immune cells infiltrate myocardial tissue during myocardial infarction and release a large number of pro-inflammatory cells, including IL-1β, IL-6, IL-17A and TNF-α (Thackeray et al., 2018; Wang et al., 2018). In addition, the inflammatory response is closely related to oxidative stress, and they regulate each other, triggering a vicious cycle and eventually leading to cardiomyocyte death and apoptosis (Joffre and Hellman, 2021). Thus, we detected whether ciprofol affects the inflammatory response and cardiomyocyte apoptosis after ISO insults. The results showed that the mRNA levels of IL-6, IL-17A and TNF-α were obviously increased after ISO insults, while ciprofol administration significantly reduced the expression of these inflammatory factors. In addition, ciprofol administration also prevented ISO-induced cardiomyocyte apoptosis *in vivo* and *in vitro*. Overall, our study identified for the first time ciprofol as a myocardial protectant against ISO-induced cardiac inflammation and cardiomyocytes.

Sirt1 is a member of the class III group of histone deacetylases and highly conserved during evolution (Tsvetkov et al., 2021). Multiple studies have shown that Sirt1 is involved in regulating a broad variety of cellular processes, including oxidative stress, the inflammatory response, apoptosis, autophagy and senescence (Liu et al., 2021c; Tsvetkov et al., 2021). Nrf2 is considered an important antioxidant sensor that restrains intracellular ROS production (Yu and Xiao, 2021). Emerging studies have identified that Sirt1 is associated with the activation of Nrf2, and both of them play a protective role in ISO-induced myocardial infarction (Liu et al., 2021d; El-Shaer et al., 2021). Herein, we evaluated the effect of ciprofol on the Sirt1/Nrf2 signaling pathway. Our data indicated that ISO insults decreased myocardial Sirt1, Nrf2, and HO-1 expression, and this effect was reversed by ciprofol treatment. In addition, Sirt1 and Nrf2 inhibition completely abolished the ciprofol-mediated anti-oxidative stress, anti-inflammation and anti-apoptosis effects, indicating that ciprofol activates the Sirt1/Nrf2 signaling pathway to protect the heart from ISO insults.

There are some limitations of the present study. First, the myocardial infarction model was established by ISO injection, which is not completely consistent with the pathogenesis of human myocardial infarction. Thus, we will use other myocardial infarction models to further demonstrate the cardioprotective role of ciprofol in future studies. Second, the exact mechanisms mediating the cardioprotective effect of ciprofol except the Sirt1/Nrf2 signaling pathway remain unknown. Thus, the exact mechanisms by which ciprofol attenuates ISO-induced myocardial infarction still require further study. Third, further research is still required so as to explore the optimal scheme of ciprofol treatment regarding time course and concentrations.

Taken together, we showed that ciprofol protects the heart against ISO-induced myocardial infarction by reducing cardiac oxidative stress, the inflammatory response and cardiomyocyte apoptosis. In addition, we found that ciprofol activates the Sirt1/Nrf2 signaling pathway to protect the heart from ISO insults. Overall, our results provide new insight into the protective mechanism of ciprofol against myocardial infarction.

## Data Availability

The raw data supporting the conclusion of this article will be made available by the authors, without undue reservation.
